# Application of natural language processing techniques to identify off-label drug usage from various online health communities

**DOI:** 10.1093/jamia/ocab124

**Published:** 2021-08-01

**Authors:** Brian Dreyfus, Anuj Chaudhary, Parth Bhardwaj, V Karthikhaa Shree

**Affiliations:** 1 Epidemiology, Bristol Myers Squibb, Princeton, New Jersey, USA; 2 Mu Sigma, Bengaluru, India

**Keywords:** Online Health Communities, off-label, natural language processing techniques, openFDA application programming interface

## Abstract

**Objective:**

Outcomes mentioned on online health communities (OHCs) by patients can serve as a source of evidence for off-label drug usage evaluation, but identifying these outcomes manually is tedious work. We have built a natural language processing model to identify off-label usage of drugs mentioned in these patient posts.

**Materials and Methods:**

Single patient posts from 4 major OHCs were considered for this study. A text classification model was built to classify the posts as either relevant or not relevant based on patient experience. The relevant posts were passed through a spelling correction tool, CSpell, and then medications and indications from these posts were identified using cTAKES (clinical Text Analysis and Knowledge Extraction System), a named entity recognition tool. Drug and indication pairs were identified using a dependency parser. Finally, if the paired indication was not mentioned on the label of the drug approved by U.S. Food and Drug Administration, it was tagged as off-label use of that drug.

**Results:**

Using this algorithm, we identified 289 off-label indications, achieving a recall of 76%.

**Conclusions:**

The method designed in this study identifies and extracts the semantic relationship between drugs and indications from demotic posts in OHCs. The results demonstrate the feasibility of using natural language processing techniques in identifying off-label drug usage across online health forums for a variety of drugs. Understanding patients’ off-label use of drugs may be able to help manufacturers innovate to better address patients’ needs and assist doctors’ prescribing decisions.

## INTRODUCTION

Off-label drug usage is the consumption of a drug for any indication not mentioned on its label, as approved by the U.S. Food and Drug Administration (FDA).^1^^,^^2^ Research from a survey of office-based physicians found that 21% of prescriptions were for off-label use.^3^ The availability of benefit-risk profiles of a medicine used off-label is limited or could be entirely unknown. Although physicians prescribing medications for off-label use may provide an opportunity for innovation, there are scant data on the safety profile of drugs used for unapproved indications.

Health authorities such as the FDA require the marketing authorization holder (MAH) to report on the off-label use of their medicines.^4^ Traditionally, the monitoring of off-label use by an MAH is derived from the spontaneous reports that are received and stored in the corporate safety database. Research has shown that spontaneous reporting of adverse events represents no more than 5% to 10% of the real incidence of events, and off-label use reports have been observed with an even lower frequency.^5^ The underreporting of off-label use adverse events, likely because of the unawareness about the possibility of reporting, as off-label use is permitted in the United States, and the fear of reporting medical errors limits the usefulness of these data sources in ascertaining the safety profile of a medicine for off-label indications.

In a chart review of patients treated with gabapentin, nearly 95% of the patients received the drug off-label, and the drug had minimal benefit.^6^ A broad analysis of free text narratives in electronic medical records (EMRs) using natural language processing (NLP) had relatively good success at detecting off-label use.^7^

Social media websites, including online health communities (OHCs), Twitter, Facebook, and others, are potentially the largest source of data related to off-label use of medications. In addition to the sheer breadth of data available from social media, these data represent most directly the patient’s voice. Existing research on OHC patient posts serve as source of evidence for off-label drug usage mentions.^8^ User posts on Reddit related to opioid addiction were analyzed and alternative unapproved treatments were found to be promoted by the patients on other online communities.^9^ For pharmaceutical companies that are the MAH of a medication, these posts present an opportunity to comprehensively evaluate the drug’s benefit-risk profile. Because the posts in OHCs are in the form of health discussions, they contain requisite information such as the drug name and indication but clinical details regarding the case such as the medical history of the patient may not be available. The NLP method in this study is based on a patient taking a specific drug for a specific indication (s). Because the posts contain conversations and discussions, the main goal was to identify and extract the requisite information that contain the relevant patient experience. Pharmacovigilance departments currently do not routinely listen to the patient’s voice via OHCs. The process of manually perusing through patient posts on online communities for off-label drug usage would be time-consuming. Also, to study these trends on a periodic basis is a tedious task.^10^ Hence, to reduce the manual effort involved, we built a model to scan patient posts to online health forums, capture relevant posts with patient experience, and identify off-label drug usage. Though online health forums provide us with huge volumes of real-world data, not all of it can be consumed, owing to the noise in the data. A major part of the methodology is dedicated to identifying posts that capture patients experience associated with a specific drug name and an indication. This analysis represents the early stages of research being conducted on data sources beyond the traditional spontaneous reports to comprehensively ascertain patients’ experiences with off-label use of medicines.

## MATERIALS AND METHODS

**Figure 1.**
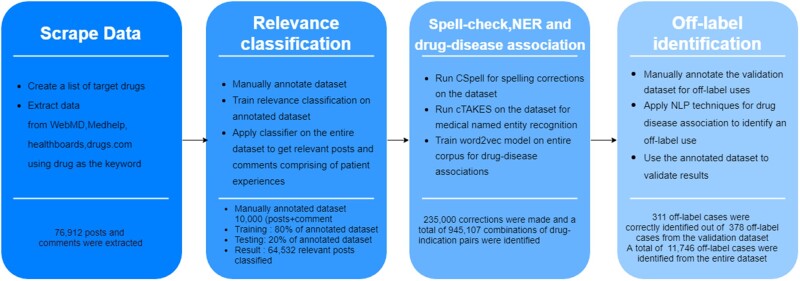
Project process flow. cTAKES: clinical Text Analysis and Knowledge Extraction System; NER: named entity recognition; NLP: natural language processing.

A diagram of the overall methods, including data collection, relevance classification, spell-check, named entity recognition (NER), drug-disease association, and off-label identification, is presented in Figure 1.

### Data

OHCs are forums where patients discuss, in the form of comments or posts, diseases and their impact, medical treatments, and drug composition. Social media forums, like blogs or social networks, including OHCs, generate huge amounts of data with a wide range of information driven by the contribution of the users sharing their experience, opinions, and acquired knowledge. Extensive exploration of OHC data represents the source of evidence for identifying off-label drug usage mentions for this study.

Taking into consideration the privacy policies and the complexities of the structure of the posts and comments on these OHCs, the following sources were selected: MedHelp, WebMD, Drugs.com, and HealthBoards.com. The analysis was conducted on a large and diverse drug list across therapeutic areas under 2 categories (positive and negative cohorts), with 6 drugs under each category. Positive cohort drugs were expected to have off-label drug usage, whereas the negative cohort drugs were not expected to have major off-label drug usage. After a review of the literature, the following drugs were selected as the positive cohort, as they were known to have off-label uses: pregabalin,^11^ gabapentin,^11^^,^^12^ lorazepam,^13^ methylphenidate,^14^ amitriptyline,^15^ and quetiapine.^16^ The negative cohort drugs considered were as follows: levonorgestrel, levofloxacin, clopidogrel, celecoxib, buprenorphine-naloxone, and natalizumab. These drugs were selected due to belonging to a therapeutic area or indication where off-label usage is difficult and unexpected based on literature review.

A total of 76 912 posts for the target drug list were scraped using Python code from the previously mentioned OHCs to form the base dataset for the analysis. The entire process can be divided into 4 major steps: (1) extract the posts from OHCs, (2) build a relevance classification model to target the posts with key information, (3) identify the medical entities like drug name and indication from the relevant posts, and (4) build a rule-based model to identify off-label drug usage using NLP techniques.

### Relevance classification model

The posts or comments on OHCs are entered firsthand by the public.^17^ These posts include knowledge about drugs, medical conditions (along with their personal experience, including general statements with no direct patient experience), and questions about the drug that do not have any information regarding consumption of the drug by patients. Comments with no information regarding the consumption of a drug did not contribute to our analysis. This analysis is focused on comments that include patient experience with a drug of interest and that mention an indication for use.^18^ Comments from patients themselves, members of their family, or friends are referred to as a patient’s personal experience. A patient’s personal experience with drug consumption or any prescription of the drug by a physician is referred to as a relevant comment in this study.

To classify a comment as relevant or not relevant, a text classification model was built based on the previous definition. The conventional method of building a text classification model^19^^,^^20^ was followed, in which the text is first converted to vectors using standard vectorization techniques (Count Vectorizer and term frequency–inverse document frequency [TF-IDF]) and also with combinations of vectorization techniques: Count Vectorizer and TF-IDF, Count Vectorizer and Doc2Vec,^21^ TF-IDF and Doc2Vec, and long short-term memory networks and convolutional neural networks. Second, these vectorization techniques were incorporated into the following models: support vector machines,^22–24^ naive Bayes, recurrent neural network, stochastic gradient descent classifier, random forest,^22–24^ and XGBoost.^24^

To train these models, 10 000 comments from the base dataset were randomly selected and manually annotated for relevance of the comment (mention of patient experience). The annotated dataset consisted of around 7000 relevant comments; the remaining 3000 comments were determined to be irrelevant. These 10 000 comments were further split into separate sets to train (80%) and test (20%) the text classification model.

The 6 vectorization techniques and their associated performance metrics are presented (by model) in Table 1. To identify the best model, we focused on F_1_ scores. The highest F_1_ score (84%; accuracy of 80%) was observed in the XGBoost model, with a vectorization ensemble of TF-IDF and Count Vectorizer. This model was further validated using k-fold cross-validation with k value as 10, which maintained consistency across folds. With the best-suited XGBoost model parameters, accuracy, precision, and recall were 83.67%, 85.76%, and 89.37%, respectively. This is the text classification model used to classify the base dataset into “relevant” and “not relevant” comments. The entire corpus of 76 912 posts was processed by this XGBoost model, yielding 64 000 posts tagged as relevant comments. This is the dataset that was examined for off-label drug usage.

**Table 1.** Classic text classification model results

Model	Vectorization Technique	Accuracy	Precision	Recall	F_1_ Score
**SVM**	**Count Vectorizer (without Stopwords)^a^**	75.46%	81.88%	76.43%	79.06%
	**Count Vectorizer^a^**	77.28%	83.41%^d^	78.03%	80.63%
	**TF-IDF (without Stopwords)^a^**	76.52%	79.88%	81.88%	80.87%
	**TF-IDF^a^**	79.16%	81.26%	85.29%	83.23%
**Naive Bayes**	**Count Vectorizer (without Stopwords)^c^**	66.40%	69.30%	78.20%	73.48%
	**Count Vectorizer^c^**	69.90%	73.70%	77.10%	75.36%
	**TF-IDF (without Stopwords)^c^**	66.30%	65.20%	93.70%^d^	76.89%
	**TF-IDF^c^**	70.10%	68.10%	94.00%^d^	78.98%
**RNN**	**Count Vectorizer (without Stopwords)^c^**	76.00%	75.00%	75.00%	75.00%
	**Count Vectorizer^c^**	79.50%^d^	79.00%	78.00%	78.50%
	**TF-IDF (without Stopwords)^c^**	75.80%	68.00%	63.00%	65.40%
	**LSTM+CNN^c^**	81.00%^d^	79.00%	78.00%	78.50%
**SGDC**	**Count Vectorizer (without Stopwords)^a^**	75.16%	82.84%	74.42%	78.40%
	**Count Vectorizer^a^**	72.73%	87.15%^d^	64.51%	74.14%
	**TF-IDF^a^**	79.41%	81.48%	85.44%	83.41%^d^
	**Count Vectorizer + Doc2Vec**	68.73%	83.28%^d^	60.56%	70.13%
	**TF-IDF + Doc2Vec**	71.85%	84.92%^d^	65.12%	73.71%
**Random Forest**	**Count Vectorizer (without Stopwords)^a^**	76.98%	78.43%	85.54%	81.83%
	**Count Vectorizer^a^**	78.53%	78.41%	89.09%^d^	83.41%^d^
	**TF-IDF (without Stopwords)^a^**	77.65%	78.21%	87.49%	82.59%
	**TF-IDF^a^**	78.40%	78.73%	88.19%^d^	83.19%
	**Count Vectorizer + TF-IDF^b^**	78.28%	78.34%	88.69%^d^	83.19%
**XGBoost**	**Count Vectorizer (without Stopwords)^a^**	78.53%	81.87%	82.93%	82.40%
	**Count Vectorizer^a^**	80.35%^d^	83.09%^d^	84.83%	83.95%^d^
	**TF-IDF (without Stopwords)^a^**	78.31%	81.49%	83.08%	82.28%
	**TF-IDF^a^**	79.92%^d^	81.69%	86.19%	83.88%^d^
	**Count Vectorizer + TF-IDF^b^**	80.16%^d^	82.12%	85.99%	84.01%^d^

CNN: convolutional neural network; LSTM: long short-term memory; RNN: recurrent neural network; SGDC: stochastic gradient descent classifier; SVM: support vector machine; TF-IDF: term frequency–inverse document frequency.

a With 900 features.

b Total 900 features with 450 features for each vectorization technique.

c With 300 features.

d Top 5 results for each metric.

### NER using clinical text analysis and the knowledge extraction system

Spelling correction is one of the fundamental segments required for most of the NLP applications. The patient posts were written by laypersons, so the health-related discussions in these posts sometimes contained irregular spellings along with orthographic errors. To standardize the language of the content and to prepare it for linguistic analysis, spelling streamlining was performed by incorporating the spell checker for consumer language (CSpell).^25^ Using CSpell, more than 235 000 words, including misspelled medical entities, were identified in the relevant patient posts dataset. A total of 1730 relevant posts with the drug of interest were identified that would have been missed because of spelling errors in the posts.

Once the language of the content was streamlined, the NER functionality of the clinical Text Analysis and Knowledge Extraction System (cTAKES) was employed to identify the medical entities mentioned in the patient posts. Recognizing the key medical entities from the relevant patient posts classified by the text classification model is a crucial process for the off-label drug usage identification. cTAKES is an open-source NLP system used for information extraction from text.^26^ By connecting to the Unified Medical Language System (UMLS) Metathesaurus database, the NER of cTAKES identifies medical entities grouped under procedures, disease disorders, signs and symptoms, and anatomy and drugs. The UMLS Metathesaurus is used as a complete knowledge source in the medical and healthcare field.^27^ By utilizing the vast concepts and relationships contained by the UMLS, extraction of useful knowledge from patient posts in OHCs is possible.

In this process of identifying medical entities, a few indications identified were observed to be nonmedical entities. To filter out such entities, Word2Vec^28^^,^^29^ embedding was implemented by calculating the cosine similarity score between the drug of interest and the indication mentioned in the comment. When the calculated similarity score returned a negative value, the identified entity was confirmed as nonmedical and removed from the final list.

### Off-label identification

**Figure 2.**
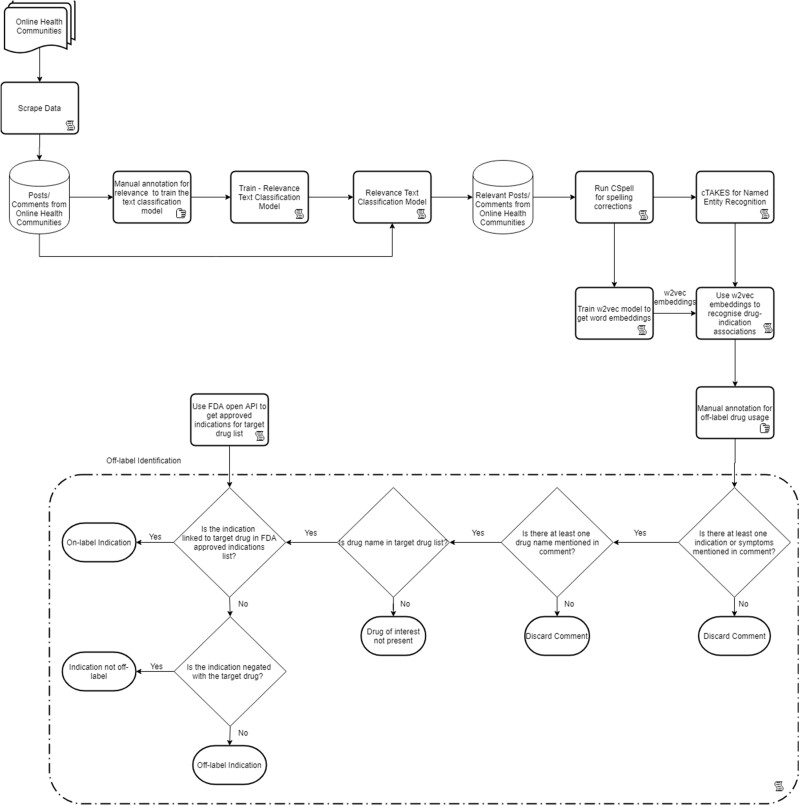
Detailed process flow for off-label identification. API: application programming interface; cTAKES: clinical Text Analysis and Knowledge Extraction System; FDA: U.S. Food and Drug Administration.

A diagram of the process for determining if a comment indicates a medication was used off-label is presented in Figure 2. The FDA-approved indications for the target drug list were extracted from the label of the drugs on the FDA’s official website,^4^ incorporating the openFDA application programming interface^30^ to create a mapping file.

A sample of 5000 random comments was taken from the 64 000 relevant comments and were manually annotated as “off-label” or “not off-label.” Comments that could not be classified as either “off-label” or “not off-label” (770 comments) were labeled as “ambiguous comments.” Comments that did not contain the necessary drug and indication information required for off-label prediction (3122 comments) were removed from the manually annotated off-label dataset. The remaining 1878 comments, which included ambiguous comments, was labeled dataset A; a subset of dataset A, which excluded the 770 ambiguous comments, was labeled dataset B.

In order to understand the interdependency of words in a comment and procure the combinations of drug-indication pairs, ScispaCy^31^ was used as the dependency parser. The result was sent to the NetworkX^32^^,^^33^ tool, which was used to generate dependency trees. As dependency parsing works on a sentence level, the process of finding links between the drug and indication also works on a sentence level across all the sentences in the comment. In an event in which the drug and indication are not mentioned in the same sentence, the preceding and succeeding sentences are checked for an indication mention. If presence of an indication is detected, the sentence is checked for the presence of a word that is being used as a substitution for the drug (it, drug, medication). On detection of a substitute word, the word is replaced with the drug name and dependency is then checked. The resultant dependency between a pair of words from the dependence tree may be erroneous when the words are too far apart in a comment. Thus, a threshold of 9 jumps between the respective pair of words, using parameter tuning, was used. If the distance between the 2 words was within this threshold, the pair was retained for further analysis.

The drug-indication pairs at the comment level were compared with the approved indication list for the target drug created using openFDA application programming interface. If the pair was not in the approved indication list, the model flagged it as off-label for that drug, otherwise was flagged as not off-label. Further, to check and remove mentions of any negations between the drug and indication, NegSpacy,^34^ a negation handling pipeline, was incorporated. Drug-indication pairs with negation involved were flagged as not off-label for that drug by the model. The result was in the form of an off-label flag (off-label or not off-label for the target drug) and confidence flag (confidence bucket of the off-label flag).

## RESULTS

A total of 76 912 posts and comments were extracted from the 4 OHCs using 12 target drugs as keywords. Of that total, 64 000 comments were classified as relevant by the XGBoost relevance classifier. After these comments were passed through our NLP-based model, a total of 11 927 posts or comments were identified as off-label by the off-label identifier. For validation, our model was tested on a gold standard dataset of 5000 manually annotated comments, resulting in recall, accuracy, and precision scores of 80%, 71%, and 40%, respectively (Table 2). The model was also tested with a subset (dataset B) of the gold standard dataset to see the model performance without any ambiguous comments (Table 3). The off-label indications observed for our target drug list are shown in Table 4. Those indications identified most frequently (at least twice) for each drug in the positive cohort are as follows: lorazepam (sleep/insomnia, pain, and panic attack), amitriptyline (migraine, pain, and anxiety), gabapentin (pain, neuropathy, and migraine), methylphenidate (depression, anxiety, and sleep/insomnia), quetiapine (sleep/insomnia, anxiety, and mood control), and pregabalin (pain).

**Table 2.** Confusion matrix, including ambiguous comments: Dataset A

		Actual Value	
		Positive (1)	Negative (0)
**Predicted Value**	**Positive (1)**	TP = 301	FP = 462
	**Negative (0)**	FN = 77	TN = 1038

Metric results were as follows: accuracy = 71%; precision = 40%; recall = 80%; F_1_ score = 52%. TN was used as indicated and correctly classified by the model; for an FN, the post was about off-label usage but was incorrectly classified by the model; for a TP, the post was about off-label usage and was correctly classified by the model; for an FP, the post was not about off-label usage and was incorrectly classified by the model.

FN: false negative; FP: false positive; TN: true negative; TP: true positive.

**Table 3.** Confusion matrix, excluding ambiguous comments: Dataset B

		Actual Value	
		Positive (1)	Negative (0)
**Predicted Value**	**Positive (1)**	TP = 301	FP = 149
	**Negative (0)**	FN = 77	TN = 581

Metric results were as follows: accuracy = 80%; precision = 67%; recall = 80%; F_1_ score = 73%. For a TN, the post was not about off-label usage and was correctly classified by the model; for an FN, the post was about off-label usage but was incorrectly classified by the model; for a TP, the post was about off-label usage and was correctly classified by the model; for an FP, the post was not about off-label usage and was incorrectly classified by the model.

FN: false negative; FP: false positive; TN: true negative; TP: true positive.

**Table 4.** Model results

Drug Name	U.S. Food and Drug Administration–Approved Indication	Off-Label Indications Identified^a^
**Positive cohort**		
Lorazepam	Anxiety, depression	Sleep disorder/insomnia (59), pain (43), panic attacks (28), addiction (13), common cold (12), headaches (11), stroke (5), tachycardia (4), chills (3), fibromyalgia (2), hyperventilating (2), epilepsy (1),
Amitriptyline	Depression	Pain (31), sleep disorder/insomnia (30), migraine (15), headaches (8), anxiety (4), stroke (3), cystitis (2), neuropathy (2)
Gabapentin	Epilepsy, postherpetic neuralgia, seizures	Pain (52), sleep disorder/insomnia (17), depression (13), neuropathy (8), numbness (7), spasms (6), migraine (5), anxiety (4), fibromyalgia (4), swelling (3), addiction (3), heartburn (2), restless legs syndrome (2), panic attacks (1), menopause (1), irritable bowel syndrome (1), carcinoma (1), gastritis (1), angina (1)
Methylphenidate	Attention-deficit/hyperactivity disorder	Depression (25), anxiety (17), fatigue (9), insomnia (6), pain (6), stress disorder (3), cancer (3), seizures (2), autism (2), posttraumatic hyperactivity (1), nausea (1), tumors (1), appetite (1), apnea (1), diabetes (1), stroke (1), spasms (1)
Quetiapine	Bipolar disorder, bipolar I and bipolar II disorder, bipolar I disorder, bipolar I disorder manic, depression, manic, mental disorders, schizophrenia	Anxiety (49), Sleep disorder/insomnia (26), mood control (21), common cold (12), pain (12), panic attacks (8), anger management (7), addiction (7), paranoia (6), nauseous (5), agitation (5), fatigue (4), hallucinations (4), dyskinesia (3), migraine (2), stroke (2), dizziness (2), palpitations (2), maniac disorder (1), allergy (1), apnea (1), psychosis (1), headaches (1), chronic fatigue syndrome (1), panicky (1), hypothyroidism (1), hives (1)
Pregabalin	Spinal cord injury, diabetic peripheral neuropathy, fibromyalgia, neuropathic pain, partial onset seizures, postherpetic neuralgia	Pain (165), headaches (10), shingles (9), migraine (7), anxiety (7), influenza (6), neuralgia (5), depression (5), arthritis (4), cancer (3), paresthesia (2), spondylitis (1)
**Negative cohort**		
Celecoxib	Acute pain, dysmenorrhea, juvenile rheumatoid arthritis, osteoarthritis, pain, primary dysmenorrhea, rheumatoid arthritis	Tendonitis (4), cancer (2), swelling (2), spasms (2), headaches (1), hematuria (1), fibromyalgia (1), carcinoma (1), sleep disorder (1)
Buprenorphine	Overdose, pain, drug abuse	Anxiety (4), depression (3), fibromyalgia (1), tumors (1)
Natalizumab	Multiple sclerosis	Infection (2), asthma (1), neuropathy (1)
Clopidogrel	Antiplatelet agent	None identified
Levonorgestrel	Contraception	None identified
Levofloxacin	Broad spectrum antibiotic	None identified

a Off-label indications are listed in decreasing order of frequency.

## DISCUSSION

In this study, we built a text classification model to apply to patient posts from 4 OHCs (MedHelp, WebMD, Drugs.com, and HealthBoards.com) to determine the extent of off-label use of 12 medications (6 for which off-label use was likely, 6 for which off-label use was unlikely). NLP of social media is increasingly being used to address public health research questions.^35^ We have designed a unique NLP algorithm that utilizes a combination of techniques to analyze OHCs for information about off-label use of medications.

Using our algorithm, relevant posts (relaying patient experience with a drug of interest and mentioning an indication) were screened for drug-indication pairs, which were then compared with the FDA label to determine if there was off-label use. The algorithm was validated using 5000 manually annotated comments (annotated and reviewed by authors), achieving an accuracy of 71%. Of 64 000 comments classified as relevant, a total of 11 927 (18.6%) posts or comments were identified as off-label. This is in good agreement with Radley et al,^3^ who found that 21% of prescriptions written by office-based physicians were off-label. A somewhat higher percentage (28.1%) of pediatric patient visits resulted in an off-label use of a medication.^36^

Though our model showcases the results for identifying off-label drug usage, it accounts only for the patient-experienced sign and symptom, regardless of it being a sign or symptom of the indication. In some cases, the entities identified as an off-label indication may be signs or symptoms of the approved indication for the drug.

Furthermore, the potential for off-label usage identification may be correlated to the classification of the drugs into positive and negative cohorts. The rationale behind using this method is to help with the validation of the algorithm. The model was expected to identify more off-label usage for drugs in the positive cohort and little to none in the negative cohort. This hypothesized outcome was successfully validated by the results. Reports from patients posted online provide a source of evidence for the use of medications for which there are no published efficacy data, but identification of these off-label uses by manual search is prohibitively time-consuming. Therefore, physicians are left to rely on their own clinical experience.^4^ The automated search of outcomes related to off-label use has the potential to generate enough evidence to warrant the initiation of traditional clinical trials. Indeed, off-label use does present a possible safety concern. A recent study of off-label prescribing for children in 2 hospitals by Lee et al^37^ found that the children who died had a higher number of off-label prescriptions than did the total cohort. The health authorities require marketing authorization holders to report off-label use of their medicines in periodic reports. This research marks a preliminary step in understanding the social media data from OHCs as a source of data source for off-label use. Further research will need to be conducted. The mining of OHCs could help provide a more complete picture of off-label use of medications.

There are other ways to estimate off-label use of medications. Andrulyte and Bjerrum^38^ identified off-label prescriptions through data mining of pharmacy servers. However, this method lacks an easy way to identify off-label use by indication. Surveys of hospital records offer another source of information about off-label prescribing,^39^ though this method is limited to inpatient prescribing and can only be performed for a limited number of hospitals. Some hospitals have developed procedures such that doctors are required to complete a request form for off-label use of a medication.^40^ Off-label prescription of medications can also be estimated from published studies.^41^

Wallach and Ross^42^ suggest that the FDA be given authority to require pharmaceutical companies to conduct clinical trials investigating the safety and efficacy of off-label uses of their medications once those uses reach a certain sales or prescribing threshold. This additional research would help ensure that the safety and efficacy off-label uses are investigated in a coordinated manner. The mining of OHCs could provide one line of evidence regarding the degree of off-label use.

## CONCLUSION

The primary goal of this study was to identify off-label drug usage from patient experience on OHCs using NLP techniques. In this communication, we have focused on the methodology and not on the metric values of the model.

Various factors, including (1) consideration of relevant patient posts with patient experience mentions, (2) streamlining the incorrect spellings in the content of the patient posts, and (3) extraction of drug names and indications mentioned in the patient posts were used for our primary analysis, and aided in identifying off-label drug usage. Because the approach we followed is a combination of machine learning and a rule-based model, along with the incorporation of various NLP techniques, we believe that our method is robust and can be confidently used for off-label drug usage identification from OHCs.

Information regarding off-label drug usage could possibly be used by drug manufacturers to guide expansion of research on their products and by authorities tasked with monitoring off-label usage. Understanding off-label use of medications could promote investment in the clinical trials needed to provide guidance regarding the safety and efficacy in new indications. However, owing to the novel nature of these data, it is premature to consider development toward new indications without additional research and evaluation with traditional methods. The scope of this work can be expanded in the future to patient age, dosage of the drug, and mode of administration of the drug, and to identify off-label usage of a wider range of drugs. Use of a consumer health vocabulary to map commonly used consumer terms with diseases or indications may lead to identification of more drug-disease associations. Extending the use of the current algorithm to real-time data sources could act as an early identification tool for off-label drug usage.

## FUNDING

The work reported in this article was funded by Bristol Myers Squibb.

## AUTHOR CONTRIBUTIONS

All authors contributed sufficiently and meaningfully to the conception and design of the project, and to drafting, editing, and revising the manuscript. All authors approved the final version for submission and agree to be accountable for all aspects of the work.
